# *ATSAS 3.0*: expanded functionality and new tools for small-angle scattering data analysis

**DOI:** 10.1107/S1600576720013412

**Published:** 2021-02-01

**Authors:** Karen Manalastas-Cantos, Petr V. Konarev, Nelly R. Hajizadeh, Alexey G. Kikhney, Maxim V. Petoukhov, Dmitry S. Molodenskiy, Alejandro Panjkovich, Haydyn D. T. Mertens, Andrey Gruzinov, Clemente Borges, Cy M. Jeffries, Dmitri I. Svergun, Daniel Franke

**Affiliations:** a European Molecular Biology Laboratory, Hamburg Site, Notkestrasse 85, Building 25 A, Hamburg, 22607, Germany; b A.V. Shubnikov Institute of Crystallography, Federal Scientific Research Centre ‘Crystallography and Photonics’ of Russian Academy of Sciences, Leninsky prospekt 59, Moscow, 119333, Russian Federation

**Keywords:** small-angle scattering, data analysis, biological macromolecules, structural modelling, *ATSAS*

## Abstract

The *ATSAS* software suite encompasses a number of programs for the processing, visualization, analysis and modelling of small-angle scattering data, with a focus on the data measured from biological macromolecules. Here, new developments in the *ATSAS 3.0* package are described. They include *IMSIM*, for simulating isotropic 2D scattering patterns; *IMOP*, to perform operations on 2D images and masks; *DATRESAMPLE*, a method for variance estimation of structural invariants through parametric resampling; *DATFT*, which computes the pair distance distribution function by a direct Fourier transform of the scattering data; *PDDFFIT*, to compute the scattering data from a pair distance distribution function, allowing comparison with the experimental data; a new module in *DATMW* for Bayesian consensus-based concentration-independent molecular weight estimation; *DATMIF*, an *ab initio* shape analysis method that optimizes the search model directly against the scattering data; *DAMEMB*, an application to set up the initial search volume for multiphase modelling of membrane proteins; *ELLLIP*, to perform quasi-atomistic modelling of liposomes with elliptical shapes; *NMATOR*, which models conformational changes in nucleic acid structures through normal mode analysis in torsion angle space; *DAMMIX*, which reconstructs the shape of an unknown intermediate in an evolving system; and *LIPMIX* and *BILMIX*, for modelling multilamellar and asymmetric lipid vesicles, respectively. In addition, technical updates were deployed to facilitate maintainability of the package, which include porting the *PRIMUS* graphical interface to Qt5, updating *SASpy* – a *PyMOL* plugin to run a subset of *ATSAS* tools – to be both Python 2 and 3 compatible, and adding utilities to facilitate mmCIF compatibility in future *ATSAS* releases. All these features are implemented in *ATSAS 3.0*, freely available for academic users at https://www.embl-hamburg.de/biosaxs/software.html.

## Abstract 2

*ATSAS* is a comprehensive software suite for the processing, visualization, analysis and modelling of small-angle scattering data. This article describes developments in the *ATSAS 3.0* release, including new programs for data simulation and for the structural modelling of lipids, nucleic acids and polydisperse systems.

## 1. Introduction   

**Figure 1:**
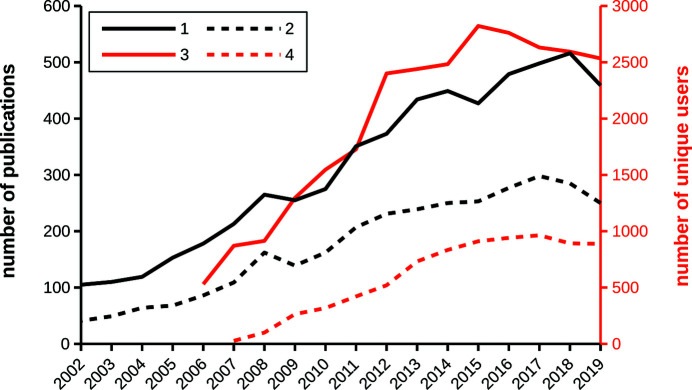
The number of biological SAS publications per year (1) has steadily increased over the past two decades, accompanied by an increase in the number of biological SAS publications which cite the *ATSAS* software suite (2). The numbers of unique users per year that downloaded *ATSAS* (3) and used the web applications in *ATSAS online* (4) also show a concurrent increase.

Small-angle scattering (SAS) of X-rays (SAXS) or neutrons (SANS) provides low-resolution structural information about various nanostructured systems, including biological macromolecules in solution (Svergun *et al.*, 2013 ▸). Over the past two decades, SAS has become an increasingly common technique in the integrative structural biology toolkit (Graewert & Svergun, 2013 ▸; Tuukkanen *et al.*, 2017 ▸; Brosey & Tainer, 2019 ▸). Importantly, SAS can be combined with high-resolution methods such as X-ray crystallography, nuclear magnetic resonance and cryo-electron microscopy, as well as other biophysical techniques like circular dichroism, static and dynamic light scattering, and cross-linking mass spectrometry (Lipfert & Doniach, 2007 ▸; Kachala *et al.*, 2015 ▸; Kikhney & Svergun, 2015 ▸; Mertens & Svergun, 2017 ▸). Solution SAS, in particular, allows the study of macromolecules in close to physiological environments and also the effects of changing environmental conditions, for example by varying temperature, pH or pressure, or by adding ligands. Increased availability of and continuous improvements to SAXS laboratory sources, third-generation synchrotrons, high-neutron-flux nuclear reactors and spallation sources have contributed to the growth of the biological SAS community (Fig. 1 ▸), which in turn has spurred developments in sample environments and instrument hardware (Classen *et al.*, 2013 ▸; Kirby *et al.*, 2013 ▸; Pernot *et al.*, 2013 ▸; Acerbo *et al.*, 2015 ▸; Blanchet *et al.*, 2015 ▸; Heller *et al.*, 2018 ▸; Liu *et al.*, 2018 ▸; Wood *et al.*, 2018 ▸). For instance, size-exclusion chromatography coupled to SAS (SEC-SAS), first demonstrated by Mathew *et al.* (2004 ▸), has now become a routine approach for the separation and structural analysis of mixture components, and is offered at many SAXS and SANS beamlines, as well as for laboratory instruments (David & Pérez, 2009 ▸; Graewert *et al.*, 2015 ▸; Jordan *et al.*, 2016 ▸; Brennich *et al.*, 2017 ▸; Yeh *et al.*, 2017 ▸; Johansen *et al.*, 2018 ▸; Ryan *et al.*, 2018 ▸; Bucciarelli *et al.*, 2018 ▸; Inoue *et al.*, 2019 ▸). Improved X-ray detectors enable time-resolved measurements at shorter timescales and, in combination with lasers and rapid mixing devices, facilitate the study of macromolecular kinetics (Cammarata *et al.*, 2008 ▸; Kubelka, 2009 ▸; Pollack, 2011 ▸; Graceffa *et al.*, 2013 ▸; Levantino *et al.*, 2015 ▸; Tuukkanen *et al.*, 2017 ▸; Josts *et al.*, 2020 ▸).

In a solution SAS experiment, the scattered radiation is generally isotropic and recorded as a 2D detector image. The isotropic data are azimuthally (‘radially’) averaged into a 1D scattering intensity curve 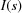, 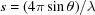, where 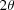 is the angle between the scattered and the incident radiation and 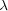 is the wavelength. Increased data quality can be achieved through the collection of replicate exposures from the sample, which are averaged into a single 1D scattering profile. Replicate exposures are similarly collected and averaged for the solvent. The resulting average is subtracted from the average sample scattering as a background, which includes the solvent, the sample holder and parasitic scattering effects (Svergun *et al.*, 2013 ▸). The background-subtracted 1D scattering profile thus represents scattering data from the sample alone and can be used to derive important structural characteristics such as the radius of gyration (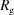) (Guinier, 1939 ▸), maximum dimension (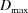), pair distance distribution function [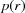] (Glatter, 1977 ▸; Svergun, 1992 ▸; Hansen, 2012 ▸), Porod volume (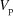) (Porod, 1951 ▸) and molecular weight (MW) (Orthaber *et al.*, 2000 ▸; Mylonas & Svergun, 2007 ▸; Rambo & Tainer, 2013 ▸; Hajizadeh *et al.*, 2018 ▸; Piiadov *et al.*, 2019 ▸). Low-resolution models may be generated *ab initio*, either as dummy-atom/residue models (Svergun, 1999 ▸; Svergun *et al.*, 2001 ▸; Franke & Svergun, 2009 ▸) or electron densities (Grant, 2018 ▸). Hybrid methods incorporating high-resolution models from other techniques such as X-ray crystallography can be applied to obtain atomistic representations of the macromolecule (Petoukhov & Svergun, 2005 ▸; Panjkovich & Svergun, 2016*a* ▸). Sample polydispersity – which may occur due to oligomer formation or intrinsic molecular flexibility or disorder – can be considered *e.g.* by modelling the solute as a mixture with defined components, each having different volume fractions (Tria *et al.*, 2015 ▸; Konarev & Svergun, 2018 ▸).

The software tools developed by the SAS community offer access to various data analysis and modelling options. These include the multipurpose packages *SASTBX* (Liu *et al.*, 2012 ▸), *BioXTAS RAW* (Hopkins *et al.*, 2017 ▸), *Sasview* (http://www.sasview.org) and *ScÅtter*, which contain utilities for data handling and analysis in the form of radial averaging of 2D detector images to 1D scattering profiles, calculation of model-independent structural parameters, SEC-SAXS data processing and deconvolution (for *BioXTAS RAW*), and model fitting and refinement (for *SASTBX*). Various specific modelling tools have also been developed. A non-exhaustive list includes *SASfit*, which constructs models using an extensive library of analytical expressions (Breßler, Kohlbrecher & Thünemann, 2015 ▸); *GenApp*, a modular infrastructure containing *SASSIE* and *US-SOMO*, for atomistic modelling which integrates hydro­dynamic information (Perkins *et al.*, 2016 ▸; Brookes *et al.*, 2016 ▸); *FoXS*, a web server for the calculation of SAXS data from atomic coordinates, which may be combined with docking (*FoXSDock*) or flexibility modelling (*MultiFoXS*) for biomolecular structures (Schneidman-Duhovny *et al.*, 2016 ▸); and *GENFIT*, *McSAS* and *X+*, which perform *ab initio* modelling accounting for shape polydispersity, primarily for soft-matter SAS but with applications to large supramolecular assemblies of biomolecules like micelles and fibrils (Spinozzi *et al.*, 2014 ▸; Bressler, Pauw & Thünemann, 2015 ▸ Ben-Nun *et al.*, 2010 ▸).

**Figure 2:**
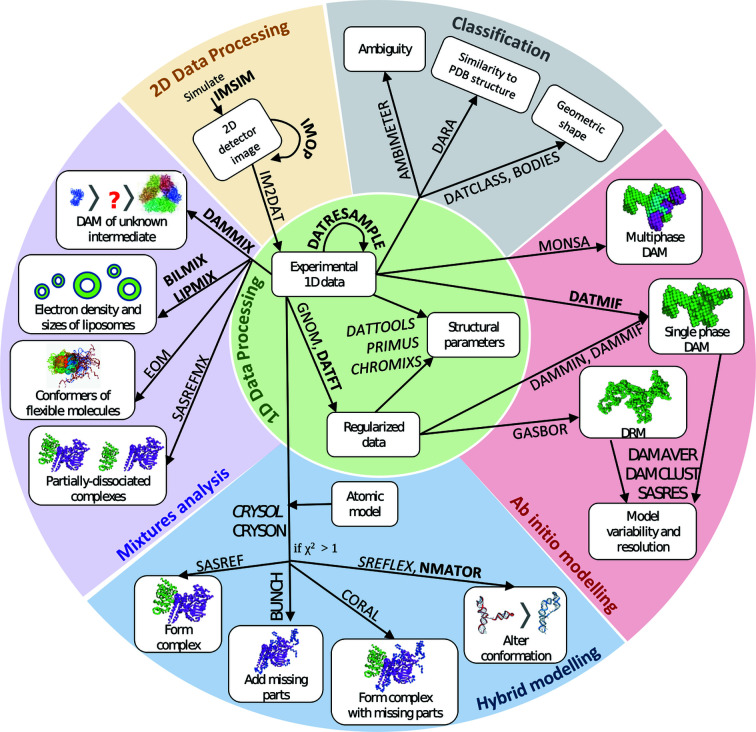
General scope of the *ATSAS* suite, including specific software for different use cases. Names in boldface indicate software newly added to *ATSAS 3.0*, while names in italics indicate updated programs (DAM: dummy-atom model; DRM: dummy-residue model).

*ATSAS* is an evolving cross-platform software suite under continuous development which encompasses numerous utilities for SAS data processing, visualization, analysis and modelling. The general scope of the *ATSAS* suite is shown in Fig. 2 ▸, which enumerates specific programs that may be used for various data analysis scenarios. The utilities are largely developed for biological solutions but are generally applicable to a wide variety of monodisperse and polydisperse systems (Konarev *et al.*, 2006 ▸; Petoukhov *et al.*, 2007 ▸, 2012 ▸; Franke *et al.*, 2017 ▸). First released in 2003, *ATSAS* has since been downloaded more than 100 000 times by over 18 000 unique users, and its use has grown along with the expanding community of biological SAS practitioners (Fig. 1 ▸). *ATSAS online*, a web application facilitating easy access to a subset of *ATSAS* tools, has similarly experienced a constant increase in usage since its release in 2007. An average of 40 000 jobs are submitted to *ATSAS online* per year, representing around 900 unique users. The growing *ATSAS* user community has served as an impetus for the continued improvement of the suite and has prompted new developments of specialized tools, several of which are discussed below. For the general *ATSAS* description we refer readers to previous publications (Konarev *et al.*, 2006 ▸; Petoukhov *et al.*, 2007 ▸, 2012 ▸) and to the comprehensive presentation of the features in *ATSAS 2.8* (Franke *et al.*, 2017 ▸). Here we focus on the changes made since the *ATSAS 2.8* release, which include major improvements in the existing tools, technological and standardization updates, new modules for data simulation, and modelling programs for specific systems such as membrane proteins, liposomes and nucleic acids.

## 2. Calculation and simulation of scattering data   

### 2.1. *CRYSOL* for anomalous SAXS   

**Figure 3:**
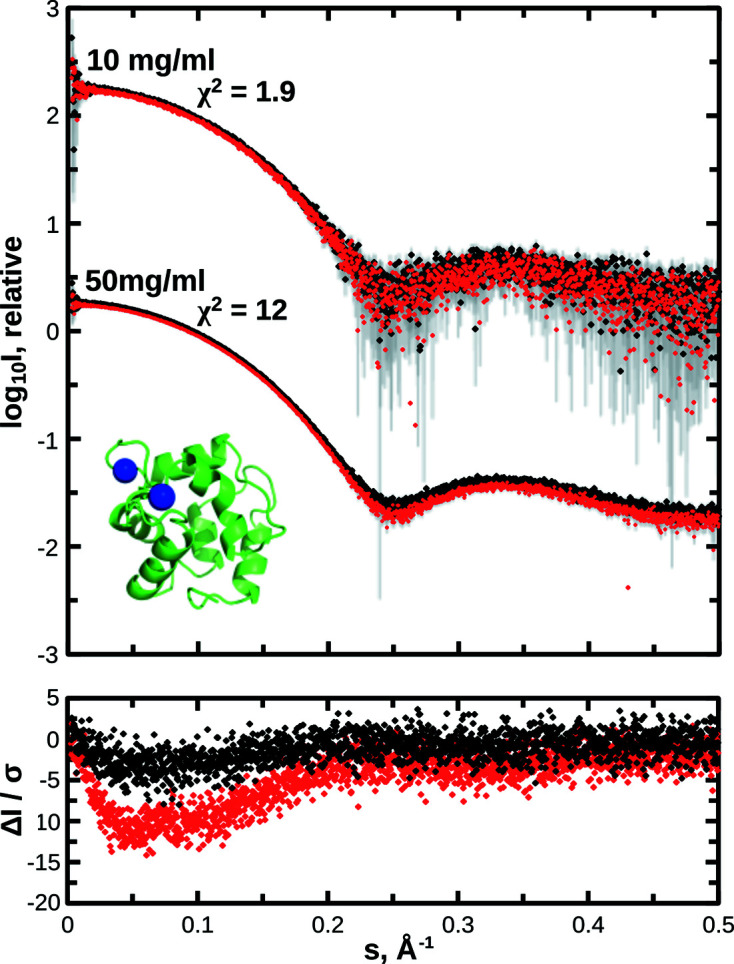
Simulated SAXS data for parvalbumin (PDB ID 1pal), with terbium atoms in two calcium-binding sites of the protein. Regular, wavelength-independent scattering (top panel, black) was computed with *CRYSOL* in default mode, while anomalous scattering (top panel, red) was evaluated with *CRYSOL* in anomalous mode, at the *L*_III_ absorption edge of terbium (7517 eV). Experimental data were simulated with *IMSIM* at two parvalbumin concentrations, 10 and 50 mg ml^−1^. *DATCMP* was used to compare regular and anomalous scattering at the two concentrations, showing greater differences at 50 mg ml^−1^ (details in Table 1 ▸). Residual plots on the bottom panel more clearly depict the differences between regular and anomalous scattering at 10 (black) and 50 mg ml^−1^ (red). At both concentrations, there is a reduction in forward scattering at the absorption edge. The difference between regular and anomalous SAXS is partly obscured by noise at 10 mg ml^−1^ but is more clearly visible at 50 mg ml^−1^ parvalbumin.

*CRYSOL* utilizes a spherical harmonics approach for rapidly calculating the scattering amplitudes and isotropic SAXS intensities from high-resolution atomic structures of macromolecules and optionally fitting the calculated scattering to experimental SAXS data (Svergun *et al.*, 1995 ▸). Since the *ATSAS 2.8* release, *CRYSOL* has been updated to provide scattering intensities not only proportional to electrons-squared units but also on an absolute scale per unit concentration [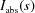 (cm^−1^)/*c* (mg ml^−1^); file extension .abs]. In addition, *CRYSOL* can now be used to calculate scattering curves that incorporate wavelength-dependent anomalous effects. Anomalous X-ray scattering occurs when the wavelength of incident radiation is at or near an atom’s absorption edge, *i.e.* at the energy that corresponds to electronic transitions of a particular element. At wavelengths close to the edge, the incident radiation is partially absorbed, resulting in electrons being excited to higher-energy states and a consequent reduction in scattering intensity (James *et al.*, 1948 ▸). This anomalous effect allows one to quantify distance information in crystallography (Hendrickson, 2014 ▸), and has also been used for the same purpose in SAXS (Stuhrmann & Notbohm, 1981 ▸; Miake-Lye *et al.*, 1983 ▸). The net reduction in the SAXS signal is, however, very low and has the potential to be lost in the background scattering (Fig. 3 ▸); therefore, accurate evaluation of the anomalous effect is of great importance in designing and cross-validating anomalous SAXS (ASAXS) experiments.

An atom’s X-ray scattering form factor *f* is represented as a function with a wavelength-independent term, 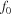, and two wavelength-dependent anomalous correction terms, 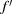 and 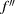 (James *et al.*, 1948 ▸):
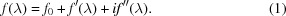
Absorption edges are wavelengths at which 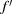 and 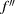 are at local minima and maxima, respectively, resulting in a decreased magnitude of the atomic form factor and an overall decrease in scattering intensity. *CRYSOL* may now be used to account for anomalous scattering effects, using the correction terms 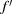 and 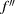 for elements from calcium to uranium, and for X-ray energies in the range from 1.0 to 29.4 keV. The corrections were tabulated by the University of Washington Biomolecular Structure Center, http://skuld.bmsc.washington.edu/scatter/AS_periodic.html. The ASAXS mode of *CRYSOL* can be accessed via the command line by specifying the absorbing element and the energy in eV. The anomalous correction terms are applied to all instances of the specified element, while the rest of the atomic form factors are computed as usual. Since the provided correction terms are theoretical and may vary from the experimental values based on the chemical environment of the absorbing atom, users may also specify custom data files containing the experimental 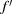 and 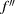 values to more accurately account for the anomalous effects.

### 2.2. Simulation of experimental scattering data   

Realistic simulated data are often required to test SAS data analysis and modelling programs on a wide variety of macromolecules, for which experimental scattering data might be unavailable. *IMSIM* (image simulation) simulates 2D SAXS patterns that can be processed into 1D scattering data using existing radial averaging applications (Franke *et al.*, 2020 ▸), *e.g.* *IM2DAT*, discussed in the next section. *IMSIM* requires calculated scattering data in absolute scale, *e.g.* from *CRYSOL*, and follows a purely statistical simulation approach, where the final intensities and error estimates of 1D patterns obtained from the radial average of the simulated 2D images exhibit the same statistical properties as observed with actual experimental data. Effects due to changes in concentration, exposure time, flux, wavelength, sample–detector distance and dimensions, pixel size, and detector mask and incident beam position can be considered in the simulation, but not systematic instrument effects. As currently implemented, *IMSIM* simulates X-ray scattering only, but with the addition of a constant to account for incoherent scattering and a resolution function to incorporate instrumental smearing effects (Barker & Pedersen, 1995 ▸) it may also be adapted to simulate 2D SANS patterns in future *ATSAS* releases.

Aside from applications in SAXS methods development and testing, the simulated data could be used, for example, to aid experimental design or beamline configuration to optimize photon counting and statistical variance in *I*(*s*), and also for educational purposes. Figs. 3 ▸ and 4 ▸ depict examples of 1D scattering profiles resulting from simulated 2D detector images from *IMSIM* which were subsequently radially averaged by *IM2DAT*.

## 3. Primary data processing   

Primary data processing spans the steps from radial averaging to the computation of model-independent structural parameters from 1D scattering data. Care should be taken in the derivation of 1D scattering data from the set of 2D detector images, particularly with the identification and removal of outlier data frames, and error estimation and propagation. The principle of ‘garbage in, garbage out’ applies here: inaccurate 1D scattering data would result in inaccurate structural parameters and potentially erroneous models. The practical implications of improper data handling are illustrated by the apparent and widespread misspecification of experimental errors in many data sets submitted to the Small-Angle Scattering Biological Databank (Kikhney *et al.*, 2020 ▸). This highlights the need for cross-validation methods, preferably at multiple steps in the data processing pipeline. Below, we discuss several updates in the *ATSAS 3.0* package which can be used for cross-validation at different processing steps, from the 2D image to the calculation of structural parameters.

### 3.1. Basic operations on 2D and 1D scattering data   

*IMOP* (image operations) is a new support application for operations on 2D images, similar to the established *DATOP* (data operations) for 1D scattering data (Franke *et al.*, 2017 ▸). *IMOP* supports addition and subtraction operations on images of equal size, as well as AND, OR and XOR operations that are intended for binary masks. In addition, it may be used to permanently apply a given bit-mask to an image. An example of the use of these elemental operations of *IMOP* is cross-validation of data reduction operations, *e.g.* by comparing radial averaging of *N* images and summing the 1D patterns versus the summation of *N* images followed by radial averaging.

*IM2DAT* (image to data), formerly called *RADAVER* (Konarev *et al.*, 2006 ▸), performs azimuthal/radial averaging of 2D detector images into 1D scattering patterns. Error estimation is based on Poisson counting statistics. To detect outliers within the data of each ring, the Poisson-distributed photon counts are transformed via the Anscombe transform (Anscombe, 1948 ▸) to approximate a normal distribution, and a median-based robust *z* score (Iglewicz & Hoaglin, 1993 ▸) is calculated to reject outliers where *z* > 4. No attempt at sub-pixel analysis (*i.e.* pixel-splitting) has been implemented as this would probably introduce correlations between neighbouring intensity estimates, which cannot easily be tracked and propagated in subsequent operations.

In contrast to past versions of *ATSAS*, in which the radial averaging application was only available upon request, *IM2DAT* has now been included by default in *ATSAS 3.0* to facilitate its use with *IMSIM*. The users may, of course, also separately employ *IM2DAT* to reprocess existing experimental 2D data. 1D data produced by radial averaging can be used for various downstream operations implemented in the *DATTOOLS* suite. Although there are no conceptual changes in *DATTOOLS* compared with its previous description (Franke *et al.*, 2017 ▸), the error propagation implemented in these tools was extensively validated and corrected where needed. Once the provenance and independence of the initial error estimates are established, they can be used in further operations.

### 3.2. Variance and residual analysis   

In SAS data analysis, several model-independent parameters, *e.g.* 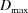, 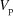 and MW, are computed as point estimates only, without an estimate of variance. In these cases, *DATRESAMPLE* may be used to determine the variability of these estimates by parametric resampling of the experimental intensities, *i.e.* by drawing randomly from a normal distribution (Marsaglia & Bray, 1964 ▸) with the expected value and standard deviation corresponding to the intensity and scaled error estimate, to account for the additional uncertainty, at each *s*. For example, to validate the 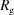 variation estimate provided by *DATRG*, a single data frame can be resampled *N* = 1000 times, with the resampling 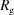 calculated for each frame from the same data range. The standard deviation of the obtained set of resampled *N* 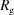 values can then be compared with the standard error estimate provided by *DATRG* for the original data. In addition to generating or validating variance estimates, *DATRESAMPLE* may be used to augment available training data for machine-learning applications by resampling a single data set *N* times.

**Figure 4:**
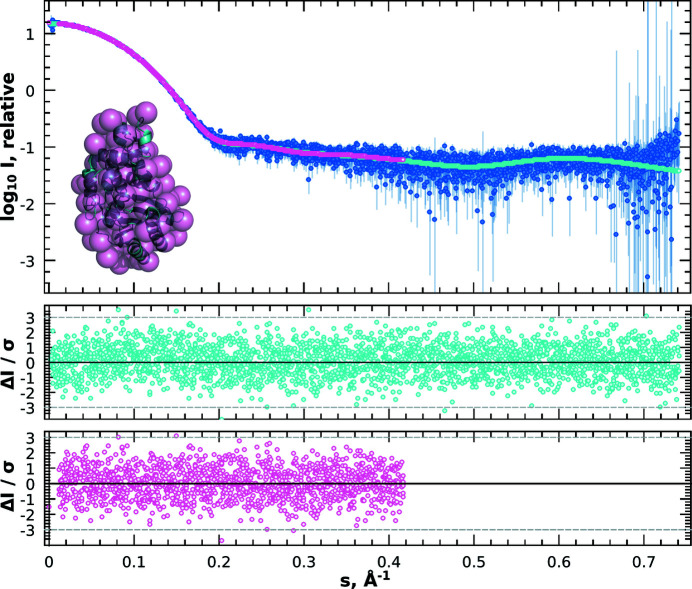
1D scattering data from beta-lactamase (PDB ID 5hw5) simulated by *IMSIM* and radially averaged with *IM2DAT* (dark blue), overlaid with the source data calculated by *CRYSOL* (cyan), and the corresponding fit of the *ab initio* model from *DATMIF* (pink). The inset shows the *DATMIF* bead model superimposed on the source model. The offset residual plots show random distribution of the residuals around zero within the expected bounds (±3). Corresponding goodness-of-fit statistics are reported in Table 1 ▸.

The analysis of the outliers allows one to identify data sets influenced by effects like sample misloading, denaturing or radiation damage. The identification of these systematic deviations is one of the most important steps in the analysis pipeline. In previous *ATSAS* releases, *DATCMP* provided two statistical tests to determine the presence of systematic deviations: the reduced 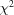 test, which requires well estimated experimental errors (Pearson, 1900 ▸), and *CORMAP*, which is independent of experimental errors (Franke *et al.*, 2015 ▸). In this release, we added the Anderson–Darling statistic to *DATCMP*. This test evaluates the goodness of fit of the distribution of standardized residuals, *i.e.* the differences between experimental data and calculated scattering, divided by the propagated error estimates, to the expected standard normal distribution (Anderson & Darling, 1954 ▸; Stephens, 1974 ▸; Marsaglia & Marsaglia, 2004 ▸). Based on the properties of the standard normal distribution, it follows that, for two SAS profiles identical up to experimental noise, the residuals should be symmetric and centred on zero, and approximately 99% of them should fall in the range of ±3 (Fig. 4 ▸). Table 1 ▸ summarizes the results of the Anderson–Darling test, alongside the reduced 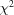 and *CORMAP* tests, for the cases illustrated in Figs. 3 ▸ and 4 ▸. The first two cases in Table 1 ▸ involve the comparison of regular and anomalous scattering curves simulated from parvalbumin [Protein Data Bank (PDB) ID 1pal; Declercq *et al.*, 1991 ▸] at two different concentrations (Fig. 3 ▸). At both concentrations, the standardized residuals were observed to have large systematic deviations from the standard normal distribution, and the hypothesis of the data sets being identical up to noise can be rejected at a significance level of 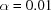 for all three *DATCMP* tests, *i.e.* anomalous scattering effects, although rather small, are still reliably detected by the statistical tests. The next two cases in Table 1 ▸ are illustrated in Fig. 4 ▸. The (arbitrarily selected) high-resolution structure of beta-lactamase (PDB ID 5hw5; Roose *et al.*, in preparation) was used as a model structure, from which noiseless scattering data were calculated using *CRYSOL* and experimental effects simulated using *IMSIM*. The *IMSIM*-simulated data were further used to generate an *ab initio* bead model with *DATMIF*. The third case in Table 1 ▸ compares the noiseless scattering data calculated with *CRYSOL* with those simulated using *IMSIM*, while the fourth compares the scattering profile of the *ab initio* bead model and the simulated data. In these last two cases, the hypothesis of being identical up to noise cannot be rejected at a significance level of 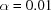 for all the tests in *DATCMP*. As illustrated by the residual plots in Fig. 4 ▸, there are no systematic deviations in either case, the standardized residuals are randomly distributed, and their distribution follows, indeed, a standard normal distribution as underlined by the Anderson–Darling test.

### 3.3. Derivation and validation of the *p*(*r*) function   

Real-space distance information can be extracted from SAS data as a pair distance distribution function, 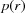. The scattering intensity 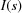 is the Fourier transform of the 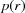 function:
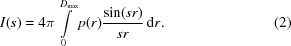
The 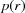 function is then derived from 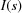 by the inverse transform:
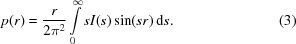



Using equation (3) to compute the 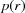 function directly from experimental data is challenging, due to the limited angular range that can be physically measured and the contribution of experimental noise, particularly at high angles. To overcome these difficulties, indirect Fourier transformation approaches were developed, such as *GNOM* in the *ATSAS* package (Svergun, 1992 ▸). Here, *p*(*r*) is parameterized by a set of analytical functions, and regularization is employed to balance the fit to experimental data and smoothness of the resulting distribution in real space, while also accounting for possible smearing effects (Glatter, 1977 ▸; Semenyuk & Svergun, 1991 ▸; Hansen, 2012 ▸). However, the direct application of equation (3) might be worth revisiting, especially as improvements in instrumentation, data collection and detector technologies have made experimental data less noisy and increasingly over-sampled, with often negligible smearing effects.

The program *DATFT* was developed to compute the 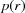 function through a direct Fourier transform of 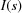, without the use of regularization. This approach is applicable if 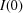 and 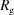 are reliably assessed from the data using the Guinier approximation (*e.g.* in the absence of aggregation and interparticle interference) and may be used to cross-validate the *p*(*r*) function obtained from *GNOM*. To reduce termination effects – artificial oscillations in the *p*(*r*) function, which are caused by the absence of scattering data at higher angles (Harris, 1978 ▸) – *DATFT* extrapolates high-angle data as 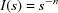, where the value of *n* can be selected (*e.g.* 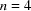 for globular particles and 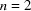 for flexible chains). As input, *DATFT* takes the experimental scattering data, the desired number of points in the 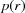 function and its distance range 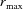. In addition, 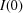 and 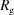 must be provided to *DATFT* to facilitate the extrapolation of truncated low-angle data using the Guinier approximation (Guinier, 1939 ▸). The resulting 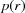 function gives an estimate of 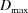, as well as 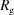 derived from the entire experimental data set, which can be used to cross-validate the 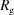 estimated from the Guinier region (*s* < 1/*R*_g_) (Feigin & Svergun, 1987 ▸). Generally, no data pre-processing is required before the application of *DATFT*. However, best results are achieved for low-noise experimental data on an equidistant *s* grid.

To verify whether the given 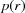 function is consistent with the experimental scattering data, a new tool, *PDDFFIT*, can be employed, which is useful for both the programs utilizing the reciprocal-space fits and those modelling directly to the *p*(*r*) function. *PDDFFIT* derives the scattering data from the 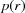 function using equation (2), allowing a convenient comparison with experimental data with *DATCMP* or *PRIMUS*/*Qt*. Two helper tools were also added to *ATSAS* for manipulating output files from *GNOM*: *OUT2POFR* and *OUT2FIT*. *OUT2POFR* extracts the 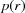 function into a separate file, *e.g.* for plotting with a third-party software application, while *OUT2FIT* does the same for the fit between the experimental data and the Fourier transform of the *p*(*r*) function.

### 3.4. Protein MW estimates from SAXS data   

MW estimates derived from solution scattering data provide important information about possible aggregation or the oligomeric state of a macromolecule in solution. SAXS-derived MW estimates can be obtained if the concentration of the macromolecule is known, by comparing against scattering either from pure water or from a reference sample of known concentration and MW (Orthaber *et al.*, 2000 ▸; Mylonas & Svergun, 2007 ▸). In the absence of accurate concentration estimates, for example for SEC-SAXS experiments, concentration-independent methods can be used. Some concentration-independent MW assessment methods use scattering invariants that are independent of data scaling, such as the Porod invariant (*Q*_p_) to obtain an estimate of the volume (*V*_p_) of the sample, from which MW is derived by dividing the volume by the partial specific volume to obtain MM_Qp_ (Porod, 1951 ▸), and applying additional corrections as done in *DATPOROD* and *SAXSMoW* (Petoukhov *et al.*, 2012 ▸; Piiadov *et al.*, 2019 ▸). Another scattering invariant, the volume of correlation (*V*_c_), was found to correlate with MW in a large survey of protein and RNA structures in the PDB, and this relationship can be used for MW estimation (Rambo & Tainer, 2013 ▸). The machine-learning method *DATCLASS* also leveraged numerous structures in the PDB, performing shape classification and *D*_max_ and MW estimation from scattering data, independent of data scaling (Franke *et al.*, 2018 ▸). In addition to the individual methods, we developed a Bayesian approach to combine the concentration-independent MW estimates into a single consensus value, while also providing a probability estimate and credibility interval (Hajizadeh *et al.*, 2018 ▸). All methods mentioned are combined into a command-line tool *DATMW*, which is also accessible from the graphical user interface *PRIMUS*/*Qt* (described in Section 5).

## 4. Structure modelling using SAS data   

SAS-based structure modelling goes beyond the parameters derived from primary data analysis to provide insight into the 3D organization of macromolecular systems. The modelling approaches for monodisperse systems range from *ab initio* methods that are purely based on the scattering data to hybrid methods incorporating high-resolution models of domains/subunits and biochemical information. Additionally, scattering data from polydisperse systems can be modelled as mixtures of several scattering species, where the SAS data allow the evaluation of their volume fractions in solution. Below we discuss new structure modelling tools in the current *ATSAS* release as well as new features added to existing tools. Of particular note are the approaches for lipid and nucleic acid structure analysis developed in response to the increased use of SAS to characterize these types of macromolecules.

### 4.1. *Ab initio* methods   

*Ab initio* modelling is applicable in cases where no structural information is available about the macromolecule of interest. *ATSAS* contains several *ab initio* modelling tools that are based on either comparison with simple shapes (*BODIES*) (Konarev *et al.*, 2003 ▸), bead/dummy-atom models (*DAMMIN*, *DAMMIF* and *MONSA*) (Svergun, 1999 ▸; Franke & Svergun, 2009 ▸; Svergun & Nierhaus, 2000 ▸), or, in the case of proteins, dummy amino-acid representations (*GASBOR*) (Svergun *et al.*, 2001 ▸). Below, we briefly describe two new tools for *ab initio* modelling in *ATSAS 3.0*.

#### 4.1.1. Direct modelling from experimental data   

Several *ab initio* bead modelling applications in the *ATSAS* suite (*DAMMIN*, *DAMMIF*, *GASBOR*) do not model the experimental data directly, using instead the regularized scattering data computed by *GNOM* during the generation of the 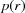 function. A new application, *DATMIF*, derived from *DAMMIF*, has been added to the current *ATSAS* release. *DATMIF* produces bead models by direct fitting of the scattering data, thereby making use of the experimental error estimates. Aside from the data fit, the only modelling penalty applied by *DATMIF* is the Akaike information criterion (AIC), which minimizes the number of parameters (in this case, beads). Hence, the AIC minimizes the volume of the final model, which results in compact protein-like structures (Fig. 4 ▸, inset).

#### 4.1.2. Multiphase modelling of solubilized membrane proteins   

**Figure 5:**
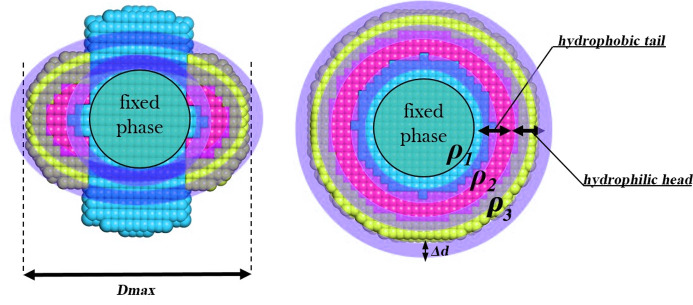
A *DAMEMB*-generated initial search volume for multiphase modelling of membrane proteins with *MONSA*. The protein phase, ρ_1_ (cyan), is defined within a spherical core region, located at the origin of the search volume. The core volume is surrounded by two distinct phases, ρ_2_ and ρ_3_, corresponding to the tail (pink) and head group (yellow) regions of a detergent molecule. The thickness of each phase, as well as that of the boundary region Δ*d*, may be specified by the user.

*MONSA* performs *ab initio* modelling of systems consisting of multiple phases with distinct contrasts (Svergun, 1999 ▸; Svergun & Nierhaus, 2000 ▸) and may thus be used to model detergent-solubilized transmembrane proteins. However, the *ab initio* reconstruction of membrane proteins is an ill-posed problem, with an even larger number of potential solutions than the single-phase *ab initio* modelling. A proper use of additional information about the system is therefore essential for this type of *ab initio* analysis. A new preparatory tool, *DAMEMB*, imposes knowledge-based constraints by building the initial *MONSA* search volume consisting of three phases corresponding to the protein, detergent tails and detergent heads (Fig. 5 ▸). Users may specify the thickness of the last two phases on the basis of the chemistry of the detergent used. To facilitate optimal data fitting in *MONSA*, the phase assignment of the boundary regions between each pair of phases is variable, including any boundary shared between the protein core and the solvent phases. *DAMEMB* may also be used for membrane-associated proteins by shifting the protein phase to the surface of the search volume, and symmetry restrictions may be imposed.

### 4.2. Hybrid methods   

Hybrid modelling methods can be employed in cases where either partial or full high-resolution structures of the macromolecule of interest are available. Hybrid methods in *ATSAS* utilize either rigid-body or flexible modelling approaches. In rigid-body methods, the high-resolution structures are represented as immutable blocks arranged in space to optimally fit the scattering data, while also meeting geometric criteria such as structure connectivity and lack of clashes. *ATSAS* programs for rigid-body modelling include, but are not limited to, *SASREF*, which models oligomers and complexes given the structures of the subunits; *BUNCH*, which builds multidomain protein models given the structures of the domains while adding missing linker residues; and *CORAL*, a combination of the above two methods, to model protein complexes with missing residues (Petoukhov & Svergun, 2005 ▸; Petoukhov *et al.*, 2012 ▸). Flexible modelling does not keep the high-resolution models fixed, instead allowing them to change conformation. For example, the *ATSAS* program *SREFLEX* permits high-resolution protein structures to be morphed along their Cartesian normal modes, in order to find alternative conformations better agreeing with the experimental scattering data (Panjkovich & Svergun, 2016*a* ▸).

In the current *ATSAS* release, two hybrid modelling tools were added: *ELLLIP*, for the rigid-body modelling of bicellar systems, and *NMATOR*, for modelling conformational changes in nucleic acid structures. Below we present these new tools, as well as updates to *SREFLEX*.

#### 4.2.1. Quasi-atomistic bicellar modelling   

**Figure 6:**
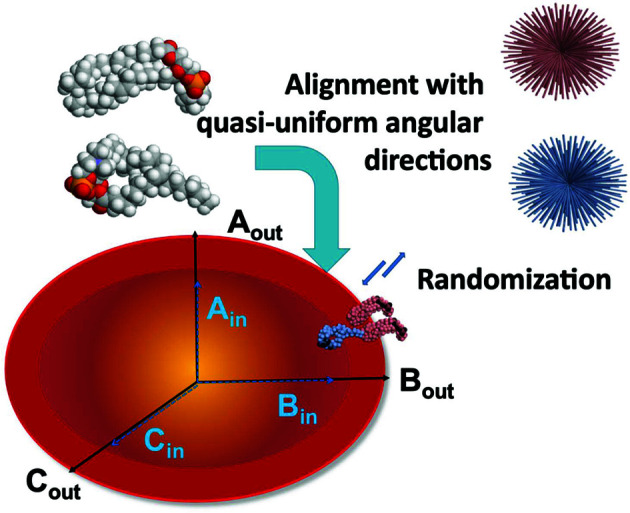
*ELLLIP* builds a liposome as two nested quasi-ellipsoids corresponding to the inner and outer liposome leaflets. The ellipsoidal shapes can be user specified by defining the lengths of the ellipsoid semi-axes (*A*_out_, *B*_out_ and *C*_out_ for the outer leaflet, and *A*_in_, *B*_in_ and *C*_in_ for the inner leaflet). Atomic models of the constituent lipids (grey beads) are placed on angular grids (top right) that define the outer (pink) and inner (blue) leaflets of the liposome. After the grids have been populated with lipids, a randomization step occurs in which the lipid molecules are displaced to account for possible disorder.

The program *ELLLIP* builds quasi-atomistic models of ellipsoidal liposomes (Fig. 6 ▸) (Petukhov *et al.*, 2020 ▸). The liposomes are constructed as two nested ellipsoids corresponding to the inner and outer leaflets. The sizes and shapes of the leaflets can be specified by the user by defining the lengths of the ellipsoid semi-axes. Two quasi-uniform angular grids are generated for the outer and inner liposomal leaflets, and each of them can have a user-defined number of directions. The angular grids are then populated with pairs of adjacent lipid molecules, which could be previously modelled with molecular dynamics as decoupled building blocks. Subsequently, *ELLLIP* may be used to randomize the positions of the lipids, whereby their centres are additionally displaced to account for the possible nonideality and disorder of the bilayer. In addition to liposome modelling, *ELLLIP* is applicable to other bicellar systems, *e.g.* those made of proteins. Note that the program does not perform any optimizations or fitting of the experimental data; it just generates the liposomal scaffolds, which can be used in subsequent modelling with other tools.

#### 4.2.2. Modelling conformational changes   

Normal mode analysis (NMA) approximates conformational changes of a macromolecule as coordinated, harmonic motions around an initial equilibrium position (Goldstein, 1950 ▸) and has been shown to approximate interdomain motions in many proteins (Tama & Sanejouand, 2001 ▸; Krebs *et al.*, 2002 ▸; Alexandrov *et al.*, 2005 ▸; Tobi & Bahar, 2005 ▸; Dobbins *et al.*, 2008 ▸; Wako & Endo, 2011 ▸). NMA is the basis for the *SREFLEX* algorithm (Panjkovich & Svergun, 2016*a* ▸), which models conformational changes in proteins by modifying an initial structure using its low-frequency normal modes in Cartesian space in the search for the model providing improved fit to experimental scattering data. *SREFLEX* can be used, for example, to model conformational differences between the crystal and solution structures, provided that these differences are detectable by SAS. A new feature has been implemented in the current version of *SREFLEX*, which produces a pool of alternative models from an initial high-resolution structure. The pool mode of *SREFLEX* can be used as a source of initial models for modelling structures with intrinsic flexibility, for example, with *EOM*, the ensemble optimization method (Tria *et al.*, 2015 ▸).

**Figure 7:**
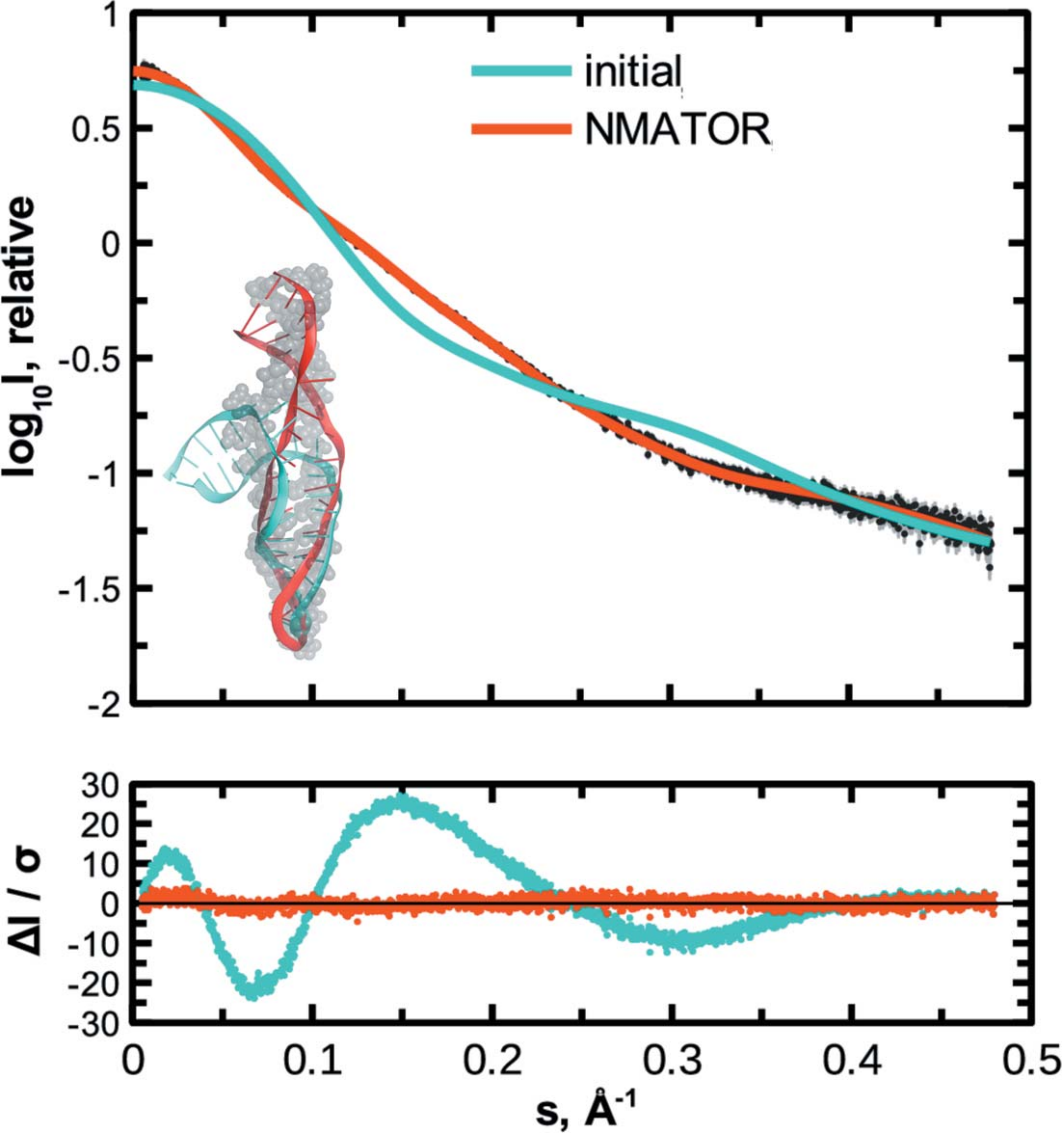
*NMATOR* models conformational changes in RNA structures to fit SAS data, while preserving bond lengths. Both the initial and target models were obtained from the solution NMR ensemble of U65 Box H/ACA snoRNA (35 nt; PDB ID 2pcv; Jin *et al.*, 2007 ▸). The target model is shown as grey spheres in the bottom-left inset, with the initial model superimposed in cyan. SAXS data were simulated from the target model with *IMSIM*. The conformational differences between the initial and target models are detected as a poor fit between the *IMSIM*-simulated SAXS data from the target and the scattering data computed by *CRYSOL* from the initial model (statistics are summarized in Table 1 ▸). The *NMATOR* model (red) recapitulated the unbending of the short helix, resulting in a better correspondence to the target model and a much better fit to the simulated data. The residuals are shown in the bottom panel.

*SREFLEX* was found to work well for proteins but has limitations for nucleic acids, possibly leading to breaks in the modified models. The new program *NMATOR* also employs NMA to capture conformational differences by SAS (Fig. 7 ▸ and Table 1 ▸) but uses the normal modes in torsion angle space instead of Cartesian space (Manalastas-Cantos & Svergun, 2021 ▸). *NMATOR* has been optimized for single-chain nucleic acid structures, morphing high-resolution models through coordinated, iterative bond rotations that alter the backbone dihedral angles: *i.e.* φ and ψ for protein structures; α, β, γ, ɛ and ζ for nucleic acids. In order to prevent spuriously large amplitudes at the ends of the molecule that may occur due to lighter packing, we have added a stiffening factor to the tip regions, as described by Lu *et al.* (2006 ▸). Since only bond rotations are imposed, *NMATOR* avoids the nonviable motions that may result from NMA in Cartesian space; the latter does not consider bond connectivity, and can thus introduce distortions due to excessive bond stretching or compression (López-Blanco & Chacón, 2016 ▸). *NMATOR* can be used in three modes: (i) to compute normal modes in torsion angle space, (ii) to refine an initial structure along its normal modes and fit the experimental SAXS data, as discussed above, and (iii) to generate a pool of alternative configurations from the initial model, which can be used for ensemble modelling of flexible structures, in a similar way to *SREFLEX*’s pool mode.

### 4.3. Polydisperse systems   

In contrast to monodisperse systems, in which all particles in solution are identical, polydisperse systems require data analysis methods that take into account both the structures and the volume fractions of different particles in solution. The scattering profile from a mixture can be represented as the volume-weighted sum of the scattering profiles of the individual components:
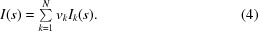
Here the mixture is assumed to contain *N* distinct scattering species, each with the scattering profile *I_k_*(*s*), comprising volume fractions *v_k_*. The addition of unknown variables to the system, such as scattering species of unknown structure and/or concentration, necessitates the use of multiple distinct scattering curves to adequately constrain the possible solutions. Depending on the type of polydisperse system, the scattering curves can either represent different time points (for evolving systems) or different sample conditions.

In the present *ATSAS* release, three new methods were added to characterize polydisperse systems: *DAMMIX*, for *ab initio* reconstruction of an unknown intermediate in an evolving system; *LIPMIX* and *BILMIX*, to model polydispersity in multilamellar and asymmetric lipid vesicles, respectively.

#### 4.3.1. Modelling evolving systems   

**Figure 8:**
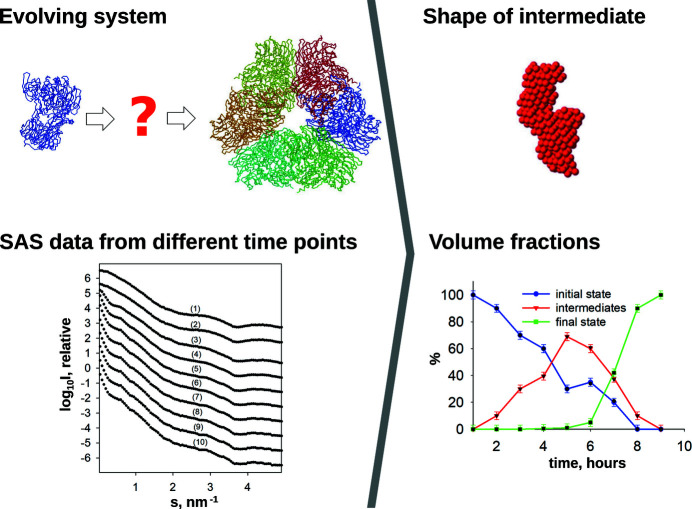
*DAMMIX* reconstructs the structure of an unknown intermediate in an evolving system, on the basis of known initial and final states, and experimental SAXS data collected at different time points. The volume fractions of the initial, intermediate and final states at each time point are also derived.

*DAMMIX* reconstructs *ab initio* the low-resolution shape of a transient component together with its volume fraction, on the basis of multiple scattering patterns recorded from an evolving system (Konarev & Svergun, 2018 ▸). The system is assumed to be a closed three-component mixture with known starting and final structures, and an unknown intermediate to be reconstructed. The three components have volume fractions with the relationship 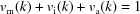, for *k* scattering curves representing different time points, where 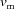, 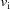 and 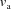 are volume fractions for the monomer (starting structure), intermediate and aggregate (final structure), respectively (Fig. 8 ▸).

*DAMMIX* can also be applied to two-component evolving systems when one component (*e.g.* the monomer) is known, allowing the reconstruction of the unknown component. In addition, *DAMMIX* can be used to retrieve the shapes of unknown components in systems with multiple assembly states, for instance, virus-like particles or nanoparticles stabilized by polymer chains. For these more complicated pathways, chemometric approaches such as multivariate curve resolution–alternating least squares (MCR-ALS) (Herranz-Trillo *et al.*, 2017 ▸) and evolving factor analysis (EFA) (Maeder, 1987 ▸; Maeder & Neuhold, 2007 ▸; Meisburger *et al.*, 2016 ▸) could aid in finding subsets of the data taken along the pathways where *DAMMIX* may be applied.

#### 4.3.2. Modelling polydisperse lipid vesicles   

**Figure 9:**
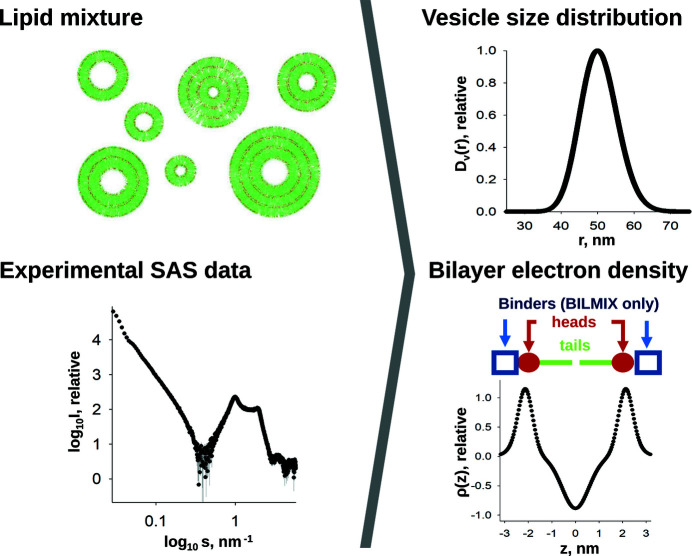
*LIPMIX* and *BILMIX* model the size distribution of liposomes [*D*_v_(*r*)] and their electron-density profiles [ρ(*z*)], based on experimental scattering data. Positioned above the ρ(*z*) plot is a schematic depicting the location in the lipid bilayer that is being represented.

The programs *BILMIX* (Konarev *et al.*, 2020 ▸) and *LIPMIX* (Konarev *et al.*, 2021 ▸) use scattering data from a mixture of lipid vesicles to reconstruct the electron density across the lipid bilayer [ρ(*z*)] and the size distribution of the vesicles [*D*_v_(*r*)] (Fig. 9 ▸). *BILMIX* can account for vesicle anisotropy, while *LIPMIX* allows the vesicles to be modelled as multilayered structures.

In both programs, the scattering data from a lipid vesicle are approximated using a separated form factor (SFF) approach. SFF is a product of the form factor of a thin spherical shell *F*_TS_, which defines the vesicle size, and the form factor of a flat lipid bilayer *F*_FB_ describing the electron density across the bilayer (Kiselev *et al.*, 2002 ▸; Pencer *et al.*, 2006 ▸). The scattering profile of each distinct vesicle *k* of a specific size and architecture can thus be expressed as
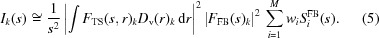



The last term in equation (5) is implemented only in *LIPMIX*, and accounts for the presence of *M* distinct multilayer architectures, each with an inter-bilayer structure factor *S_i_* ^FB^ and occupancy factor *w_i_* (Zhang *et al.*, 1994 ▸).

The form factor *F*_FB_(*s*) is the Fourier transform of the electron-density profile ρ(*z*) (Fig. 9 ▸, right panel), defined as
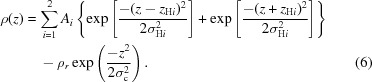
The two Gaussian terms of width σ_H1_, centred at ±*z*_H1_, represent the hydro­philic head groups, while the Gaussian term of width σ_c_ centred at *z* = 0 (the middle of the bilayer) represents the electron density of the hydro­phobic core. The two Gaussian terms of width σ_H2_, centred at ±*z*_H2_, are implemented only in *BILMIX* and allow the modelling of asymmetric electron-density profiles, *e.g.* proteins associated with the inner or outer leaflets of the liposome. Both *BILMIX* and *LIPMIX* can be utilized to model various liposomal systems and serve as tools for lipidomics structural studies.

## 5. Technical updates and standardization   

Several changes have been made in *ATSAS 3.0* to facilitate maintainability and future development. These include preparations for read and write compatibility with the mmCIF format, as well as updates to the graphical frameworks.

### 5.1. mmCIF compatibility   

A number of programs in the *ATSAS* suite make use of high-resolution structure files, including *CRYSOL*, which computes scattering from atomic coordinates, and the hybrid modelling methods, which use high-resolution structures as building blocks for SAS-guided modelling. As the PDB has made mmCIF the new standard format for structure files (Hall & McMahon, 2005 ▸; Berman *et al.*, 2014 ▸; Adams *et al.*, 2019 ▸), the *ATSAS* software is currently being adapted to be read and write compatible with both PDB and mmCIF formats. As of the current release, the programs *BUNCH* and *NMATOR* utilize both PDB and mmCIF formats as input. In order to use *BUNCH*, a preparatory program, *PRE_BUNCH*, must first be run. This produces a single PDB file containing the domains and the appropriate number of dummy atoms representing the missing loop regions, which is then used by *BUNCH* as input. *PRE_BUNCH* has been updated to read both PDB and mmCIF structure files, thus allowing *BUNCH* to be used with mmCIFs as the initial input. For other relevant *ATSAS* applications and in the interim period while not all *ATSAS* programs are natively mmCIF compatible, a format conversion utility *CIF2PDB* can be used. *CIF2PDB* converts structure files from mmCIF to the PDB format, making them readable by all *ATSAS* programs.

### 5.2. Updates to graphical interfaces   

*PRIMUS*/*Qt* provides an interactive graphical user interface for many *ATSAS* applications and acts as an interactive plotting and data analysis tool. In the current release, *PRIMUS*/*Qt* was ported to utilize the most recent long-term support release of the Qt5 framework (https://www.qt.io) for continued and improved cross-platform support. The functional enhancements in *PRIMUS*/*Qt* include, but are not limited to, improved plot display, configurability and export to bitmap and vector graphic formats with variable size and resolution, addition of residual plots where data fitting is performed, and a redesign of the pairwise comparisons of data sets view. The latter now allows for minor mismatches of the angular grid and provides a square heatmap-like overview of comparison results employing *CORMAP* or the reduced 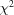 test. Further, all statistics implemented in *DATCMP* are immediately accessible in this view.

**Figure 10:**
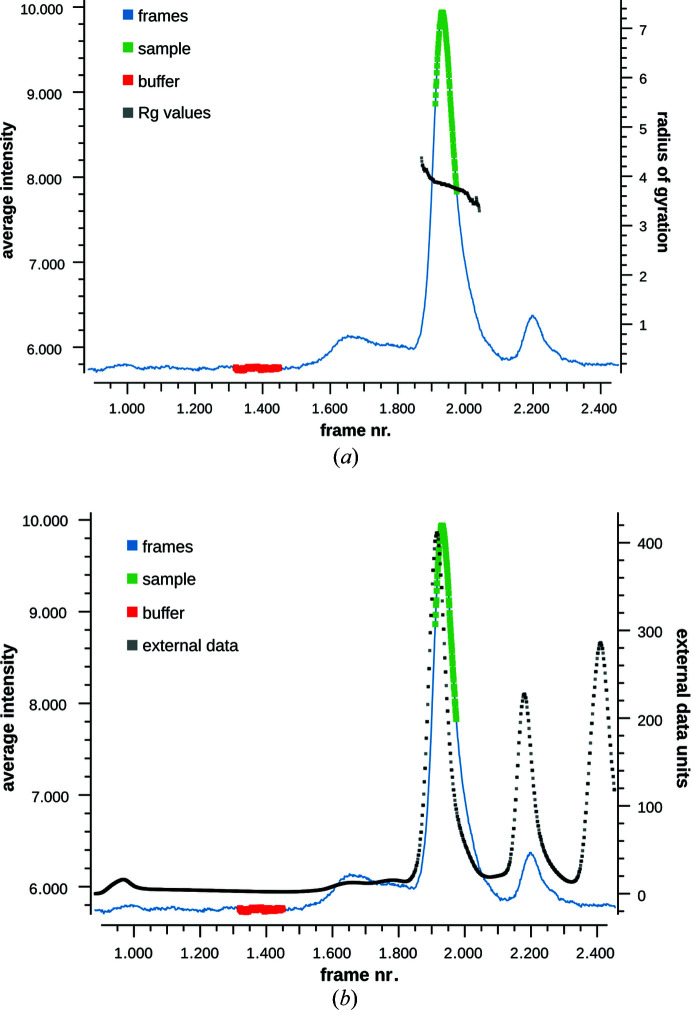
*CHROMIXS* updates. (*a*) Regions in the SEC-SAS data (blue line) which represent the sample (green, on the peak) and buffer (red, on the flat region) can be selected manually or automatically. The *R*_g_ or MW across the sample region (black correlation, through the sample elution peak) can be calculated. (*b*) Complementary time-course data (black dots), such as a UV absorbance trace to track protein elution, can be loaded and viewed together with the SEC-SAS data. The third (rightmost) UV absorbance peak corresponds to buffer mismatch, *i.e.* components in the sample buffer that are not present in the SEC running buffer.

The graphical interface in *CHROMIXS* enables a convenient and rapid display of thousands of SEC-SAS data frames, as well as manual or automated selection of sample and buffer frames (Panjkovich & Svergun, 2018 ▸). Extra features have been added to *CHROMIXS* since the *ATSAS 2.8* release, which include the calculation of MW and 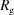 estimates for the selected sample-peak elution frames, as well as the ability to load and visualize other time-course data, *e.g.* UV absorbance (Fig. 10 ▸).

A plugin, *SASpy*, enables the usage of a subset of *ATSAS* functions within the molecular visualization system *PyMOL*, facilitating creation, manipulation and SAS-guided refinement of hybrid models in a graphical environment (Panjkovich & Svergun, 2016*b* ▸). *SASpy* has been updated to be both Python 2 and Python 3 compatible. Also, feature updates to several main *ATSAS* programs are now available in *SASpy*, such as an explicit hydrogens toggle for *CRYSOL*, which enables users to generate accurate scattering amplitudes for the input structure files with atomic groups not recognized in the default mode. *SASpy* is distributed both as a component of the *ATSAS* package and as an open-source *PyMOL* plugin (https://github.com/emblsaxs/saspy).

## 6. Conclusions   

The *ATSAS 3.0* release introduces a set of new functionalities, which include modelling tools for lipids and nucleic acids, and expanded options for polydisperse systems. Data simulation tools have also been introduced in this release, with the intention of spurring SAS methods development in a wider developer community. In addition, to facilitate maintainability and future development, *ATSAS* was updated to technical standards, including support of the mmCIF format and utilization of the most recent versions of graphical frameworks.

*ATSAS* can be installed and used locally (installers for Windows, Mac OS and Linux available at https://www.embl-hamburg.de/biosaxs/software.html). Alternatively, many programs can be run on the EMBL Hamburg cluster via the *ATSAS online* interface (https://www.embl-hamburg.de/biosaxs/atsas-online/). Feedback from the user community serves as an important guide to future developments in *ATSAS* and can be given at the SAXIER forum (https://www.saxier.org/forum/).

## Figures and Tables

**Table 1:** Summary of statistical analysis using the three *DATCMP* methods to assess goodness of fit of the data presented in Figs. 3 ▸, 4 ▸ and 7 ▸ The test values are given, with the corresponding *p* values in parentheses. For all three tests, the hypothesis that there are no significant differences between data sets holds for significance level α = 0.01 when the *p* value > α.

	*CORMAP*		Reduced χ^2^		Anderson–Darling	
Comparison	Test value	*p* value	Test value	*p* value	Test value	*p* value
Simulated† regular versus ASAXS data for 10 mg ml^−1^ parvalbumin (*n* = 2652)	117	<10^−6^	1.888	<10^−6^	455	<10^−6^
Simulated† regular versus ASAXS data for 50 mg ml^−1^ parvalbumin (*n* = 2652)	713	<10^−6^	11.970	<10^−6^	5809	<10^−6^
Noiseless‡ versus simulated† data for beta-lactamase (*n* = 2652)	13	0.2759	1.007	0.3942	0.320	0.9226
*DATMIF* model versus simulated† data for beta-lactamase (*n* = 1434)	11	0.5031	1.008	0.4188	0.611	0.6374
NMR structure of U65 Box H/ACA snoRNA (PDB ID 2pcv, model 4) versus simulated† data for same molecule, different conformation (PDB ID 2pcv, model 3) (*n* = 1776)	395	<10^−6^	18.037	<10^−6^	2398	<10^−6^
*NMATOR* model versus simulated† data for U65 Box H/ACA snoRNA (PDB ID 2pcv, model 3) (*n* = 1776)	13	0.1942	1.081	0.0091	2.284	0.0644

† From *IMSIM*.

‡ From *CRYSOL*.

## References

[bb1] Acerbo, A. S., Cook, M. J. & Gillilan, R. E. (2015). *J. Synchrotron Rad.* **22**, 180–186. 10.1107/S1600577514020360 PMC4294029 25537607

[bb2] Adams, P. D., Afonine, P. V., Baskaran, K., Berman, H. M., Berrisford, J., Bricogne, G., Brown, D. G., Burley, S. K., Chen, M., Feng, Z., Flensburg, C., Gutmanas, A., Hoch, J. C., Ikegawa, Y., Kengaku, Y., Krissinel, E., Kurisu, G., Liang, Y., Liebschner, D., Mak, L., Markley, J. L., Moriarty, N. W., Murshudov, G. N., Noble, M., Peisach, E., Persikova, I., Poon, B. K., Sobolev, O. V., Ulrich, E. L., Velankar, S., Vonrhein, C., Westbrook, J., Wojdyr, M., Yokochi, M. & Young, J. Y. (2019). *Acta Cryst.* D**75**, 451–454. 10.1107/S2059798319004522 PMC6465986 30988261

[bb3] Alexandrov, V., Lehnert, U., Echols, N., Milburn, D., Engelman, D. & Gerstein, M. (2005). *Protein Sci.* **14**, 633–643. 10.1110/ps.04882105 PMC2279292 15722444

[bb4] Anderson, T. W. & Darling, D. A. (1954). *J. Am. Stat. Assoc.* **49**, 765–769.

[bb5] Anscombe, F. J. (1948). *Biometrika*, **35**, 246–254.

[bb6] Barker, J. G. & Pedersen, J. S. (1995). *J. Appl. Cryst.* **28**, 105–114.

[bb7] Ben-Nun, T., Ginsburg, A., Székely, P. & Raviv, U. (2010). *J. Appl. Cryst.* **43**, 1522–1531.

[bb8] Berman, H. M., Kleywegt, G. J., Nakamura, H. & Markley, J. L. (2014). *J. Comput. Aided Mol. Des.* **28**, 1009–1014. 10.1007/s10822-014-9770-y PMC4196035 25062767

[bb9] Blanchet, C. E., Spilotros, A., Schwemmer, F., Graewert, M. A., Kikhney, A., Jeffries, C. M., Franke, D., Mark, D., Zengerle, R., Cipriani, F., Fiedler, S., Roessle, M. & Svergun, D. I. (2015). *J. Appl. Cryst.* **48**, 431–443. 10.1107/S160057671500254X PMC4379436 25844078

[bb10] Brennich, M. E., Round, A. R. & Hutin, S. (2017). *J. Vis. Exp. JOVE*, https://doi.org/10.3791/54861. 10.3791/54861 PMC5409194 28117806

[bb11] Breßler, I., Kohlbrecher, J. & Thünemann, A. F. (2015). *J. Appl. Cryst.* **48**, 1587–1598. 10.1107/S1600576715016544 PMC4603274 26500467

[bb12] Bressler, I., Pauw, B. R. & Thünemann, A. F. (2015). *J. Appl. Cryst.* **48**, 962–969. 10.1107/S1600576715007347 PMC4453982 26089769

[bb13] Brookes, E., Vachette, P., Rocco, M. & Pérez, J. (2016). *J. Appl. Cryst.* **49**, 1827–1841. 10.1107/S1600576716011201 PMC5045733 27738419

[bb14] Brosey, C. A. & Tainer, J. A. (2019). *Curr. Opin. Struct. Biol.* **58**, 197–213. 10.1016/j.sbi.2019.04.004 PMC6778498 31204190

[bb15] Bucciarelli, S., Midtgaard, S. R., Nors Pedersen, M., Skou, S., Arleth, L. & Vestergaard, B. (2018). *J. Appl. Cryst.* **51**, 1623–1632. 10.1107/S1600576718014462 PMC6276278 30546289

[bb16] Cammarata, M., Levantino, M., Schotte, F., Anfinrud, P. A., Ewald, F., Choi, J., Cupane, A., Wulff, M. & Ihee, H. (2008). *Nat. Methods*, **5**, 881–886. 10.1038/nmeth.1255 PMC3159148 18806790

[bb17] Classen, S., Hura, G. L., Holton, J. M., Rambo, R. P., Rodic, I., McGuire, P. J., Dyer, K., Hammel, M., Meigs, G., Frankel, K. A. & Tainer, J. A. (2013). *J. Appl. Cryst.* **46**, 1–13. 10.1107/S0021889812048698 PMC3547225 23396808

[bb18] David, G. & Pérez, J. (2009). *J. Appl. Cryst.* **42**, 892–900.

[bb400] Declercq, J. P., Tinant, B., Parello, J. & Rambaud, J. (1991). *J. Mol. Biol.* **220**, 1017–1039. 10.1016/0022-2836(91)90369-h 1880797

[bb19] Dobbins, S. E., Lesk, V. I. & Sternberg, M. J. E. (2008). *Proc. Natl Acad. Sci. USA*, **105**, 10390–10395. 10.1073/pnas.0802496105 PMC2475499 18641126

[bb20] Feigin, L. A. & Svergun, D. I. (1987). *Structure Analysis by Small-Angle X-ray and Neutron Scattering*. New York: Plenum Press.

[bb21] Franke, D., Hajizadeh, N. R. & Svergun, D. I. (2020). *J. Appl. Cryst.* **53**, 536–539. 10.1107/S1600576720000527 PMC7133063 32280325

[bb22] Franke, D., Jeffries, C. M. & Svergun, D. I. (2015). *Nat. Methods*, **12**, 419–422. 10.1038/nmeth.3358 25849637

[bb23] Franke, D., Jeffries, C. M. & Svergun, D. I. (2018). *Biophys. J.* **114**, 2485–2492. 10.1016/j.bpj.2018.04.018 PMC6129182 29874600

[bb24] Franke, D., Petoukhov, M. V., Konarev, P. V., Panjkovich, A., Tuukkanen, A., Mertens, H. D. T., Kikhney, A. G., Hajizadeh, N. R., Franklin, J. M., Jeffries, C. M. & Svergun, D. I. (2017). *J. Appl. Cryst.* **50**, 1212–1225. 10.1107/S1600576717007786 PMC5541357 28808438

[bb25] Franke, D. & Svergun, D. I. (2009). *J. Appl. Cryst.* **42**, 342–346. 10.1107/S0021889809000338 PMC5023043 27630371

[bb26] Glatter, O. (1977). *J. Appl. Cryst.* **10**, 415–421.

[bb27] Goldstein, H. (1950). *Classical Mechanics*. Reading: Addison-Wesley-Longman.

[bb28] Graceffa, R., Nobrega, R. P., Barrea, R. A., Kathuria, S. V., Chakravarthy, S., Bilsel, O. & Irving, T. C. (2013). *J. Synchrotron Rad.* **20**, 820–825. 10.1107/S0909049513021833 PMC3795536 24121320

[bb29] Graewert, M. A., Franke, D., Jeffries, C. M., Blanchet, C. E., Ruskule, D., Kuhle, K., Flieger, A., Schäfer, B., Tartsch, B., Meijers, R. & Svergun, D. I. (2015). *Sci. Rep.* **5**, 10734. 10.1038/srep10734 PMC5377070 26030009

[bb30] Graewert, M. A. & Svergun, D. I. (2013). *Curr. Opin. Struct. Biol.* **23**, 748–754. 10.1016/j.sbi.2013.06.007 23835228

[bb31] Grant, T. D. (2018). *Nat. Methods*, **15**, 191–193. 10.1038/nmeth.4581 29377013

[bb32] Guinier, A. (1939). *Ann. Phys.* **11**, 161–237.

[bb33] Hajizadeh, N. R., Franke, D., Jeffries, C. M. & Svergun, D. I. (2018). *Sci. Rep.* **8**, 7204. 10.1038/s41598-018-25355-2 PMC5940760 29739979

[bb34] Hall, S. R. & McMahon, B. (2005). Editors. *International Tables for Crystallography*, Vol. G, *Definition and Exchange of Crystallographic Data*. Dordrecht: Springer.

[bb35] Hansen, S. (2012). *J. Appl. Cryst.* **45**, 566–567.

[bb36] Harris, F. J. (1978). *Proc. IEEE*, **66**, 51–83.

[bb37] Heller, W. T., Cuneo, M., Debeer-Schmitt, L., Do, C., He, L., Heroux, L., Littrell, K., Pingali, S. V., Qian, S., Stanley, C., Urban, V. S., Wu, B. & Bras, W. (2018). *J. Appl. Cryst.* **51**, 242–248.

[bb38] Hendrickson, W. A. (2014). *Q. Rev. Biophys.* **47**, 49–93. 10.1017/S0033583514000018 PMC4128195 24726017

[bb39] Herranz-Trillo, F., Groenning, M., van Maarschalkerweerd, A., Tauler, R., Vestergaard, B. & Bernadó, P. (2017). *Structure*, **25**, 5–15. 10.1016/j.str.2016.10.013 27889205

[bb40] Hopkins, J. B., Gillilan, R. E. & Skou, S. (2017). *J. Appl. Cryst.* **50**, 1545–1553. 10.1107/S1600576717011438 PMC5627684 29021737

[bb41] Iglewicz, B. & Hoaglin, D. (1993). *The ASQC Basic References in Quality Control Statistical Techniques*, Vol. 16. ASQ Press.

[bb42] Inoue, R., Nakagawa, T., Morishima, K., Sato, N., Okuda, A., Urade, R., Yogo, R., Yanaka, S., Yagi-Utsumi, M., Kato, K., Omoto, K., Ito, K. & Sugiyama, M. (2019). *Sci. Rep.* **9**, 12610. 10.1038/s41598-019-48911-w PMC6717197 31471544

[bb43] James, R. W., Bragg, S. L. & Bragg, W. L. (1948). *The Optical Principles of the Diffraction of X-rays*. London: Bell & Sons.

[bb401] Jin, H., Loria, J. P. & Moore, P. B. (2007). *Mol. Cell*, **26**, 205–215. 10.1016/j.molcel.2007.03.014 17466623

[bb44] Johansen, N. T., Pedersen, M. C., Porcar, L., Martel, A. & Arleth, L. (2018). *Acta Cryst.* D**74**, 1178–1191. 10.1107/S2059798318007180 30605132

[bb45] Jordan, A., Jacques, M., Merrick, C., Devos, J., Forsyth, V. T., Porcar, L. & Martel, A. (2016). *J. Appl. Cryst.* **49**, 2015–2020. 10.1107/S1600576716016514 PMC5139991 27980509

[bb46] Josts, I., Gao, Y., Monteiro, D. C. F., Niebling, S., Nitsche, J., Veith, K., Gräwert, T. W., Blanchet, C. E., Schroer, M. A., Huse, N., Pearson, A. R., Svergun, D. I. & Tidow, H. (2020). *Structure*, **28**, 348–354.e3. 10.1016/j.str.2019.12.001 31899087

[bb47] Kachala, M., Valentini, E. & Svergun, D. I. (2015). *Intrinsically Disordered Proteins Studied by NMR Spectroscopy*, pp. 261–289. Cham: Springer.

[bb48] Kikhney, A. G., Borges, C. R., Molodenskiy, D. S., Jeffries, C. M. & Svergun, D. I. (2020). *Protein Sci.* **29**, 66–75. 10.1002/pro.3731 PMC6933840 31576635

[bb49] Kikhney, A. G. & Svergun, D. I. (2015). *FEBS Lett.* **589**, 2570–2577. 10.1016/j.febslet.2015.08.027 26320411

[bb50] Kirby, N. M., Mudie, S. T., Hawley, A. M., Cookson, D. J., Mertens, H. D. T., Cowieson, N. & Samardzic-Boban, V. (2013). *J. Appl. Cryst.* **46**, 1670–1680.

[bb51] Kiselev, M. A., Lesieur, P., Kisselev, A. M., Lombardo, D. & Aksenov, V. L. (2002). *Appl. Phys. Mater. Sci. Process.* **74**, s1654–s1656.

[bb200] Konarev, P. V., Gruzinov, A. Y., Mertens, H. D. T &. Svergun, D. I. (2021). *J. Appl. Cryst.* **54**, 169–179. 10.1107/S1600576720015368 PMC7941313 33833646

[bb52] Konarev, P. V., Petoukhov, M. V., Dadinova, L. A., Fedorova, N. V., Volynsky, P. E., Svergun, D. I., Batishchev, O. V. & Shtykova, E. V. (2020). *J. Appl. Cryst.* **53**, 236–243.

[bb53] Konarev, P. V., Petoukhov, M. V., Volkov, V. V. & Svergun, D. I. (2006). *J. Appl. Cryst.* **39**, 277–286.

[bb54] Konarev, P. V. & Svergun, D. I. (2018). *IUCrJ*, **5**, 402–409. 10.1107/S2052252518005900 PMC6038953 30002841

[bb55] Konarev, P. V., Volkov, V. V., Sokolova, A. V., Koch, M. H. J. & Svergun, D. I. (2003). *J. Appl. Cryst.* **36**, 1277–1282.

[bb56] Krebs, W. G., Alexandrov, V., Wilson, C. A., Echols, N., Yu, H. & Gerstein, M. (2002). *Proteins*, **48**, 682–695. 10.1002/prot.10168 12211036

[bb57] Kubelka, J. (2009). *Photochem. Photobiol. Sci.* **8**, 499–512. 10.1039/b819929a 19337664

[bb58] Levantino, M., Yorke, B. A., Monteiro, D. C. F., Cammarata, M. & Pearson, A. R. (2015). *Curr. Opin. Struct. Biol.* **35**, 41–48. 10.1016/j.sbi.2015.07.017 26342489

[bb59] Lipfert, J. & Doniach, S. (2007). *Annu. Rev. Biophys. Biomol. Struct.* **36**, 307–327. 10.1146/annurev.biophys.36.040306.132655 17284163

[bb60] Liu, G., Li, Y., Wu, H., Wu, X., Xu, X., Wang, W., Zhang, R. & Li, N. (2018). *J. Appl. Cryst.* **51**, 1633–1640.

[bb61] Liu, H., Hexemer, A. & Zwart, P. H. (2012). *J. Appl. Cryst.* **45**, 587–593.

[bb62] López-Blanco, J. R. & Chacón, P. (2016). *Curr. Opin. Struct. Biol.* **37**, 46–53. 10.1016/j.sbi.2015.11.013 26716577

[bb63] Lu, M., Poon, B. & Ma, J. (2006). *J. Chem. Theory Comput.* **2**, 464–471. 10.1021/ct050307u PMC3133770 21760758

[bb64] Maeder, M. (1987). *Anal. Chem.* **59**, 527–530.

[bb65] Maeder, M. & Neuhold, Y.-M. (2007). *Practical Data Analysis in Chemistry*. Elsevier.

[bb150] Manalastas-Cantos, K. & Svergun, D. I. (2021). In preparation.

[bb66] Marsaglia, G. & Bray, T. A. (1964). *SIAM Rev.* **6**, 260–264.

[bb67] Marsaglia, G. & Marsaglia, J. (2004). *J. Stat. Soft.* **9**, 1–5.

[bb68] Mathew, E., Mirza, A. & Menhart, N. (2004). *J. Synchrotron Rad.* **11**, 314–318. 10.1107/S0909049504014086 15211037

[bb69] Meisburger, S. P., Taylor, A. B., Khan, C. A., Zhang, S., Fitzpatrick, P. F. & Ando, N. (2016). *J. Am. Chem. Soc.* **138**, 6506–6516. 10.1021/jacs.6b01563 PMC4896396 27145334

[bb70] Mertens, H. D. T. & Svergun, D. I. (2017). *Arch. Biochem. Biophys.* **628**, 33–41. 10.1016/j.abb.2017.05.005 PMC5553349 28501583

[bb71] Miake-Lye, R. C., Doniach, S. & Hodgson, K. O. (1983). *Biophys. J.* **41**, 287–292. 10.1016/S0006-3495(83)84440-3 PMC1329182 6838970

[bb72] Mylonas, E. & Svergun, D. I. (2007). *J. Appl. Cryst.* **40**, s245–s249.

[bb73] Orthaber, D., Bergmann, A. & Glatter, O. (2000). *J. Appl. Cryst.* **33**, 218–225.

[bb74] Panjkovich, A. & Svergun, D. I. (2016*a*). *Phys. Chem. Chem. Phys.* **18**, 5707–5719. 10.1039/c5cp04540a 26611321

[bb75] Panjkovich, A. & Svergun, D. I. (2016*b*). *Bioinformatics*, **32**, 2062–2064. 10.1093/bioinformatics/btw071 PMC4920112 27153695

[bb76] Panjkovich, A. & Svergun, D. I. (2018). *Bioinformatics*, **34**, 1944–1946. 10.1093/bioinformatics/btx846 PMC5972624 29300836

[bb77] Pearson, K. (1900). *London Edinb. Dubl. Philos. Mag. J. Sci.* **50**, 157–175.

[bb78] Pencer, J., Krueger, S., Adams, C. P. & Katsaras, J. (2006). *J. Appl. Cryst.* **39**, 293–303.

[bb79] Perkins, S. J., Wright, D. W., Zhang, H., Brookes, E. H., Chen, J., Irving, T. C., Krueger, S., Barlow, D. J., Edler, K. J., Scott, D. J., Terrill, N. J., King, S. M., Butler, P. D. & Curtis, J. E. (2016). *J. Appl. Cryst.* **49**, 1861–1875. 10.1107/S160057671601517X PMC5139988 27980506

[bb80] Pernot, P., Round, A., Barrett, R., De Maria Antolinos, A., Gobbo, A., Gordon, E., Huet, J., Kieffer, J., Lentini, M., Mattenet, M., Morawe, C., Mueller-Dieckmann, C., Ohlsson, S., Schmid, W., Surr, J., Theveneau, P., Zerrad, L. & McSweeney, S. (2013). *J. Synchrotron Rad.* **20**, 660–664. 10.1107/S0909049513010431 PMC3943554 23765312

[bb81] Petoukhov, M. V., Franke, D., Shkumatov, A. V., Tria, G., Kikhney, A. G., Gajda, M., Gorba, C., Mertens, H. D. T., Konarev, P. V. & Svergun, D. I. (2012). *J. Appl. Cryst.* **45**, 342–350. 10.1107/S0021889812007662 PMC4233345 25484842

[bb83] Petoukhov, M. V., Konarev, P. V., Kikhney, A. G. & Svergun, D. I. (2007). *J. Appl. Cryst.* **40**, s223–s228.

[bb84] Petoukhov, M. V. & Svergun, D. I. (2005). *Biophys. J.* **89**, 1237–1250. 10.1529/biophysj.105.064154 PMC1366608 15923225

[bb82] Petukhov, M. V., Konarev, P. V., Dadinova, L. A., Fedorova, N. V., Volynsky, P. E., Svergun, D. I., Batishchev, O. V. & Shtykova, E. V. (2020). *Crystallogr. Rep.* **65**, 258–263.

[bb85] Piiadov, V., Ares de Araújo, E., Oliveira Neto, M., Craievich, A. F. & Polikarpov, I. (2019). *Protein Sci.* **28**, 454–463. 10.1002/pro.3528 PMC6319763 30371978

[bb86] Pollack, L. (2011). *Biopolymers*, **95**, 543–549. 10.1002/bip.21604 21328311

[bb87] Porod, G. (1951). *Colloid Polym. Sci.* **124**, 83–114.

[bb88] Rambo, R. P. & Tainer, J. A. (2013). *Nature*, **496**, 477–481. 10.1038/nature12070 PMC3714217 23619693

[bb89] Ryan, T. M., Trewhella, J., Murphy, J. M., Keown, J. R., Casey, L., Pearce, F. G., Goldstone, D. C., Chen, K., Luo, Z., Kobe, B., McDevitt, C. A., Watkin, S. A., Hawley, A. M., Mudie, S. T., Samardzic Boban, V. & Kirby, N. (2018). *J. Appl. Cryst.* **51**, 97–111.

[bb90] Schneidman-Duhovny, D., Hammel, M., Tainer, J. A. & Sali, A. (2016). *Nucleic Acids Res.* **44**, W424–W429. 10.1093/nar/gkw389 PMC4987932 27151198

[bb91] Semenyuk, A. V. & Svergun, D. I. (1991). *J. Appl. Cryst.* **24**, 537–540.

[bb92] Spinozzi, F., Ferrero, C., Ortore, M. G., De Maria Antolinos, A. & Mariani, P. (2014). *J. Appl. Cryst.* **47**, 1132–1139. 10.1107/S1600576714005147 PMC4038801 24904247

[bb93] Stephens, M. A. (1974). *J. Am. Stat. Assoc.* **69**, 730–737.

[bb94] Stuhrmann, H. B. & Notbohm, H. (1981). *Proc. Natl Acad. Sci. USA*, **78**, 6216–6220. 10.1073/pnas.78.10.6216 PMC349009 6947224

[bb95] Svergun, D. I. (1992). *J. Appl. Cryst.* **25**, 495–503.

[bb96] Svergun, D. I. (1999). *Biophys. J.* **76**, 2879–2886. 10.1016/S0006-3495(99)77443-6 PMC1300260 10354416

[bb97] Svergun, D., Barberato, C. & Koch, M. H. J. (1995). *J. Appl. Cryst.* **28**, 768–773.

[bb98] Svergun, D. I., Koch, M. H. J., Timmins, P. A. & May, R. P. (2013). *Small-Angle X-ray and Neutron Scattering from Solutions of Biological Macromolecules*. Oxford University Press.

[bb99] Svergun, D. I. & Nierhaus, K. H. (2000). *J. Biol. Chem.* **275**, 14432–14439. 10.1074/jbc.275.19.14432 10799526

[bb100] Svergun, D. I., Petoukhov, M. V. & Koch, M. H. J. (2001). *Biophys. J.* **80**, 2946–2953. 10.1016/S0006-3495(01)76260-1 PMC1301478 11371467

[bb101] Tama, F. & Sanejouand, Y.-H. (2001). *Protein Eng.* **14**, 1–6. 10.1093/protein/14.1.1 11287673

[bb102] Tobi, D. & Bahar, I. (2005). *Proc. Natl Acad. Sci. USA*, **102**, 18908–18913. 10.1073/pnas.0507603102 PMC1323175 16354836

[bb103] Tria, G., Mertens, H. D. T., Kachala, M. & Svergun, D. I. (2015). *IUCrJ*, **2**, 207–217. 10.1107/S205225251500202X PMC4392415 25866658

[bb104] Tuukkanen, A. T., Spilotros, A. & Svergun, D. I. (2017). *IUCrJ*, **4**, 518–528. 10.1107/S2052252517008740 PMC5619845 28989709

[bb105] Wako, H. & Endo, S. (2011). *Biophys. Chem.* **159**, 257–266. 10.1016/j.bpc.2011.07.004 21807453

[bb106] Wood, K., Mata, J. P., Garvey, C. J., Wu, C.-M., Hamilton, W. A., Abbeywick, P., Bartlett, D., Bartsch, F., Baxter, P., Booth, N., Brown, W., Christoforidis, J., Clowes, D., d’Adam, T., Darmann, F., Deura, M., Harrison, S., Hauser, N., Horton, G., Federici, D., Franceschini, F., Hanson, P., Imamovic, E., Imperia, P., Jones, M., Kennedy, S., Kim, S., Lam, T., Lee, W. T., Lesha, M., Mannicke, D., Noakes, T., Olsen, S. R., Osborn, J. C., Penny, D., Perry, M., Pullen, S. A., Robinson, R. A., Schulz, J. C., Xiong, N. & Gilbert, E. P. (2018). *J. Appl. Cryst.* **51**, 294–314.

[bb107] Yeh, Y.-Q., Liao, K.-F., Shih, O., Shiu, Y.-J., Wu, W.-R., Su, C.-J., Lin, P.-C. & Jeng, U.-S. (2017). *J. Phys. Chem. Lett.* **8**, 470–477. 10.1021/acs.jpclett.6b02722 28067527

[bb108] Zhang, R., Suter, R. M., Nagle, J. F. & Hansen, S. (1994). *Phys. Rev. E*, **45**, 566–567.

